# Smoking and risk of restless leg syndrome: a systematic review, meta-analysis, and Mendelian randomisation

**DOI:** 10.7189/jogh.16.04067

**Published:** 2026-03-20

**Authors:** Dongru Du, Jiangyue Qin, Xiaoju Tang, Lijuan Gao, Yanqiu Wu, Zhenni Chen, Fangying Chen, Fengming Luo, Yongchun Shen

**Affiliations:** 1 Department of Pulmonary and Critical Care Medicine, West China Hospital, Sichuan University, Chengdu, China; 2 Laboratory of Pulmonary Immunology and Inflammation, Frontiers Science Center for Disease-Related Molecular Network, West China Hospital, Sichuan University, Chengdu, China; 3 Department of General Practice, General Practice Medical Center, West China Hospital, Sichuan University, Chengdu, China; 4 Division of Pulmonary Diseases, State Key Laboratory of Biotherapy of China, Chengdu, China; 5 Department of Medical Affairs, West China Hospital, Sichuan University, Chengdu, China; 6 Department of Tuberculosis, The Third People's Hospital of Tibet Autonomous Region, Lhasa, Tibet, China

## Abstract

**Background:**

Although the link between smoking and various sleep disorders has been well-established, it is still unknown whether smoking increases the risk of restless legs syndrome (RLS). We investigated this association using a meta-analysis and explored the causality through Mendelian randomisation (MR).

**Methods:**

We searched six databases for studies reporting associations between smoking and RLS in overall adults, and the results were presented as odds ratios (ORs) with 95% confidence intervals (CIs). We performed sensitivity, subgroup and meta-regression analyses to identify potential sources of heterogeneity. We obtained data used in MR analyses from the UK Biobank and the Genome-wide Association Studies Catalogue. We applied the inverse-variance weighted method, MR Egger, weighted median, simple mode and weighted mode for data analyses, and further conducted pleiotropy and heterogeneity tests, as well as leave-one-out analyses.

**Results:**

Based on 30 studies, we found that smoking was associated with increased risk of RLS (OR = 1.40; 95% CI = 1.17, 1.67, *P* < 0.001), with the risk significantly increased (*P* = 0.04) in pregnant women (OR = 2.41; 95% CI = 1.39, 4.16, *P* = 0.002) than in the non-pregnant adults (OR = 1.30, 95% CI = 1.09, 1.55, *P* = 0.004), and in current smokers compared with former smokers (OR = 1.09; 95% CI = 1.02, 1.16, *P* = 0.01). We identified multi-centre studies, diagnostic criteria for RLS and participants' age as potential sources of heterogeneity; however, MR results did not show any causal association between smoking and RLS (OR = 0.50; 95% CI = 0.16, 1.56, *P* = 0.23).

**Conclusions:**

Although the meta-analysis suggested that smoking increases the risk of RLS, MR analyses did not provide evidence for a causal relationship. Future studies are needed to elucidate the biological mechanisms underlying this association.

**Registration:**

PROSPERO: CRD420251048406.

Restless leg syndrome (RLS) is a sleep-related movement disorder characterised by an irresistible urge to move legs, with symptoms worsening at rest or in the evening [1]. Its prevalence was reported to reach 1–3% in Asia and 5–13% in North America and Europe, exerting a detrimental impact on patients' quality of life [2]. Some of the previous studies have identified potential genetic variants of RLS, which may provide insights into its pathogenesis [3,4]. However, the association between lifestyle-related factors and RLS are still inadequately understood, posing challenges for RLS prevention and management [2,5]. Therefore, further investigations into preventable lifestyle-related risk factors of RLS are needed to address this issue.

Around 32.6% of the male population and 6.5% of the female population had a history of smoking worldwide in 2020 [6], which has been identified as a risk factor for multiple diseases, including chronic respiratory diseases, cardiovascular diseases and cancers [7–9]. Recent studies have suggested that smoking could also serve as a contributing risk factor for multiple sleep disorders, including obstructive sleep apnoea [10] and insomnia [11]. Chronic tobacco smoking was associated with increased light sleep stages (N1 and N2) and reduced slow-wave sleep compared with non-smokers, which could be partly restored after smoking cessation [12,13]. However, it is still conflicting whether smoking increases the risk of RLS [14].

Therefore, we conducted a meta-analysis to explore the association between smoking and RLS in real-world settings. We then performed a Mendelian randomisation (MR) to investigate the causality of the smoking-RLS association. The comparison of results from both approaches may refine the understanding of their relationship.

## METHODS

We registered this systematic review in PROSPERO (CRD420251048406), and report our findings per the PRISMA and Journal of Global Health’s GRABDROP guidelines **(**Table S1 in the **Online Supplementary Document)** [15,16].

### Literature search and study selection

We searched PubMed, Web of Science, Scopus, Wanfang and China National Knowledge Infrastructure for eligible studies from database inception to 30 April 2025 and updated it on 5 November 2025. We have also searched medRxiv for preprints or grey literature (Table S2 in the **Online Supplementary Document)**. We also checked the references of related reviews to identify any missing records.

We included observational studies with available data on smokers and non-smokers with or without RLS. These studies should focus on the overall adult population, including non-pregnant adults and pregnant women. We excluded studies based on the same source of data, review, meta-analysis or comments, and studies subject to selection bias, such as focusing on populations with specific diseases.

### Data extraction and quality assessments

Two investigators independently extracted baseline characteristics data from eligible studies, including the first author, publication year, geographical location of study population, study type, single-center or multi-center design and diagnostic criteria for RLS; smokers and non-smokers with or without RLS; detailed information on smokers, such as the number of former smokers and current smokers; baseline information of study population, including mean age, proportion of males, body mass index, history of hypertension, diabetes mellitus, coronary artery disease, stroke and anaemia.

As for quality assessments, we evaluated cross-sectional studies using the Agency for Healthcare Research and Quality checklist, with a total score of 11, categorising study quality as high (8–11), moderate (4–7), and low (0–3) [17]. We evaluated cohort studies using the Newcastle-Ottawa Scale, with a total score of 9, categorising them as high, moderate, and low quality for scores of 7–9, 4–6 and 0–3, respectively [18]. We excluded all studies with low quality scores from further analyses.

### Statistical analyses of meta-analysis

We calculated odds ratios (ORs) with 95% confidence intervals (CIs) to evaluate whether smoking was associated with increased risk of RLS. Since we enrolled both non-pregnant adults and pregnant women, we conducted a separate stratified analysis to address this association in both groups, respectively. We performed further meta-analyses to evaluate whether current smokers were associated with increased risk of RLS compared with former smokers, and presented the results as ORs and 95% CIs.

We performed heterogeneity assessments using the Q-test and *I*^2^ statistic. When *I*^2^>50% or *P* < 0.1 in the Q-test, we applied a random-effect model; otherwise, we used a fixed-effect model. We conducted sensitivity, subgroup and meta-regression analyses to identify the underlying source of heterogeneity, and assessed publication bias via funnel plots and asymmetry tests. We performed all statistical analyses in *R*, version 4.4.1 (R Core Team, Vienna, Austria).

### Three fundamental assumptions of MR

**Figure 1:**
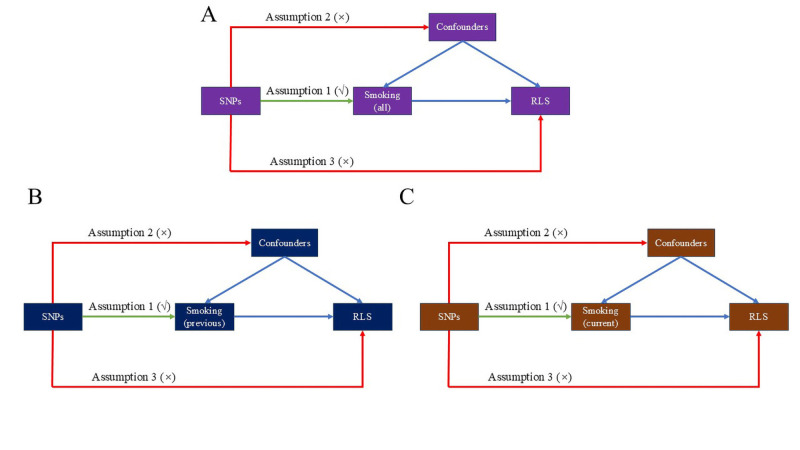
Design of Mendelian randomisation.

MR is an approach utilising single-nucleotide polymorphisms (SNPs) as instrumental variables (IVs) to assess the causal association between exposure and outcome [19]. We conducted a two-sample MR to explore whether ever smoking, former smoking, and current smoking were causally associated with RLS. To ensure the reliability of MR results, the IVs should be strongly associated with the exposure, independent of influence from any potential confounders, and could affect the outcome through exposure only [20] (**Figure 1**).

### Data source and selection of SNPs

We minimised genetic heterogeneity of the analysis by selecting only individuals of European ancestry. We obtained data on all smoking, former smoking, and current smoking from the UK Biobank, with a sample size of 336 067, 424 960 and 462 434, respectively [21]. We obtained the data of RLS from the FinnGen cohort [64], with a sample size of 453 733. We set *P <* 5 × 10^−8^ as the threshold for selection of eligible SNPs. We evaluated linkage disequilibrium with a window size of r^2^ = 0.001 and kb = 10 000, and calculated F statistics to ensure the strength of each SNP, enrolling only those with an F>10 for further analysis.

### Statistical analyses of MR

We applied the inverse variance weighted (IVW) approach as the main method to evaluate whether ever, former and current smoking causally increased the risk of RLS. We also applied MR Egger, weighted median, simple mode and weighted mode, to improve the reliability of results. We conducted an MR Egger regression to evaluate potential horizontal pleiotropy and Cochran’s Q test for heterogeneity assessments. We performed leave-one-out analyses to evaluate the stability of results and identify outliers. We conducted all analyses in *R*, version 4.4.1.

## RESULTS

### Study selection and quality assessments

We identified 897 records from six databases. After removing duplicates and irrelevant studies, we selected 79 studies for full-text review, of which we included 30, comprising 364 744 participants (108 678 smokers and 256 066 non-smokers) in the meta-analysis (Figure S1 in the **Online Supplementary Document**) [22–51]. We summarised the baseline characteristics of all eligible studies, including 25 studies focusing on the non-pregnant adults and five studies on pregnant women (**Table 1**). We rated all eligible studies as moderate to high quality based on the scoring system described above.

**Table 1:** Baseline characteristics of eligible studies

Sources	Continent	Pregnant women	Diagnostic criteria of RLS	Number of participants	Number of smokers	Number of non-smokers	Quality
Vafaei *et al.*, 2025 [22]	Asia	No	Other questionnaires	364	160	204	Moderate
Karadeniz *et al.*, 2023 [23]	Asia	Yes	IRLSSG 2012	268	25	243	Moderate
AlShareef *et al.*, 2023 [24]	Asia	No	IRLSSG 2012	8774	1312	7462	Moderate
Mubeen *et al.*, 2022 [25]	Asia	Yes	IRLSSG 2012	478	66	412	High
Kim *et al.*, 2022 [26]	Asia	No	Other questionnaires	4000	1862	2138	Moderate
Zhuang *et al.*, 2021 [27]	Asia	No	IRLSSG 2003	90 337	21 824	68 513	Moderate
Aksoy *et al.*, 2021 [28]	Asia	No	IRLSSG 2012	622	349	273	High
Zofarhari *et al.*, 2020 [29]	North America	No	IRLSSG 2003	26 304	12 284	14 020	Moderate
Huang *et al.*, 2020 [30]	Asia	No	IRLSSG 2003	1888	312	1576	Moderate
Almeneessier *et al.*, 2020 [31]	Asia	Yes	IRLSSG 2012	742	18	724	Moderate
Esposito *et al.*, 2019 [32]	Europe	Yes	IRLSSG 2003	648	113	535	Moderate
Düz *et al.*, 2019 [33]	Asia	No	IRLSSG 2012	573	180	393	Moderate
Didriksen *et al.*, 2019 [34]	Europe	No	Other questionnaires	25 336	3327	22 009	Moderate
Liu *et al.*, 2018 [35]	Asia	No	IRLSSG 2003	5324	2,643	2681	Moderate
Sherbin *et al.*, 2017 [36]	Asia	No	IRLSSG 2003	2071	425	1646	Moderate
Fereshtehnejad 2017 *et al.*, [37]	Asia	No	IRLSSG 2003	19 176	1977	17 199	Moderate
Safak *et al.*, 2016 [38]	Asia	No	IRLSSG 2003	664	248	416	High
Wali *et al.*, 2015 [39]	Asia	No	IRLSSG 2003	2682	462	2220	Moderate
Mahmood *et al.*, 2015 [40]	Asia	No	IRLSSG 2003	390	29	361	Moderate
Catzín-Kuhlmann *et al.*, 2015 [41]	South America	No	IRLSSG 2003	51 263	10 777	40 486	Moderate
Winter *et al.*, 2013 [42]	North America	No	Other questionnaires	22 786	10 750	12 036	Moderate
Innes *et al.*, 2013 [43]	North America	No	IRLSSG 2003	1209	632	577	Moderate
Hübner *et al.*, 2013 [44]	Europe	Yes	IRLSSG 2003	501	58	443	Moderate
Li 2012 *et al.*, [45]	Asia	No	IRLSSG 2003	2101	543	1558	Moderate
Batool-Anwar *et al.*, 2011 [46]	North America	No	IRLSSG 2003	65 544	22 132	43 412	Moderate
Gao *et al.*, 2010 [47]	North America	No	IRLSSG 2003	23 119	13 017	10 102	Moderate
Benediktsdottir *et al.*, 2010 [48]	Europe	No	IRLSSG 2003	1344	791	553	Moderate
Schlesinger *et al.*, 2009 [49]	Europe	No	IRLSSG 2003	1537	566	971	Moderate
Winkelman *et al.*, 2008 [50]	North America	No	IRLSSG 2003	3433	1722	1711	Moderate
Rangarajan *et al.*, 2007 [51]	Europe	No	IRLSSG 2003	1266	74	1192	Moderate

RLS – restless leg syndrome, IRLSSG – International Restless Legs Syndrome Study Group

### Smoking and risk of RLS

**Figure 2:**
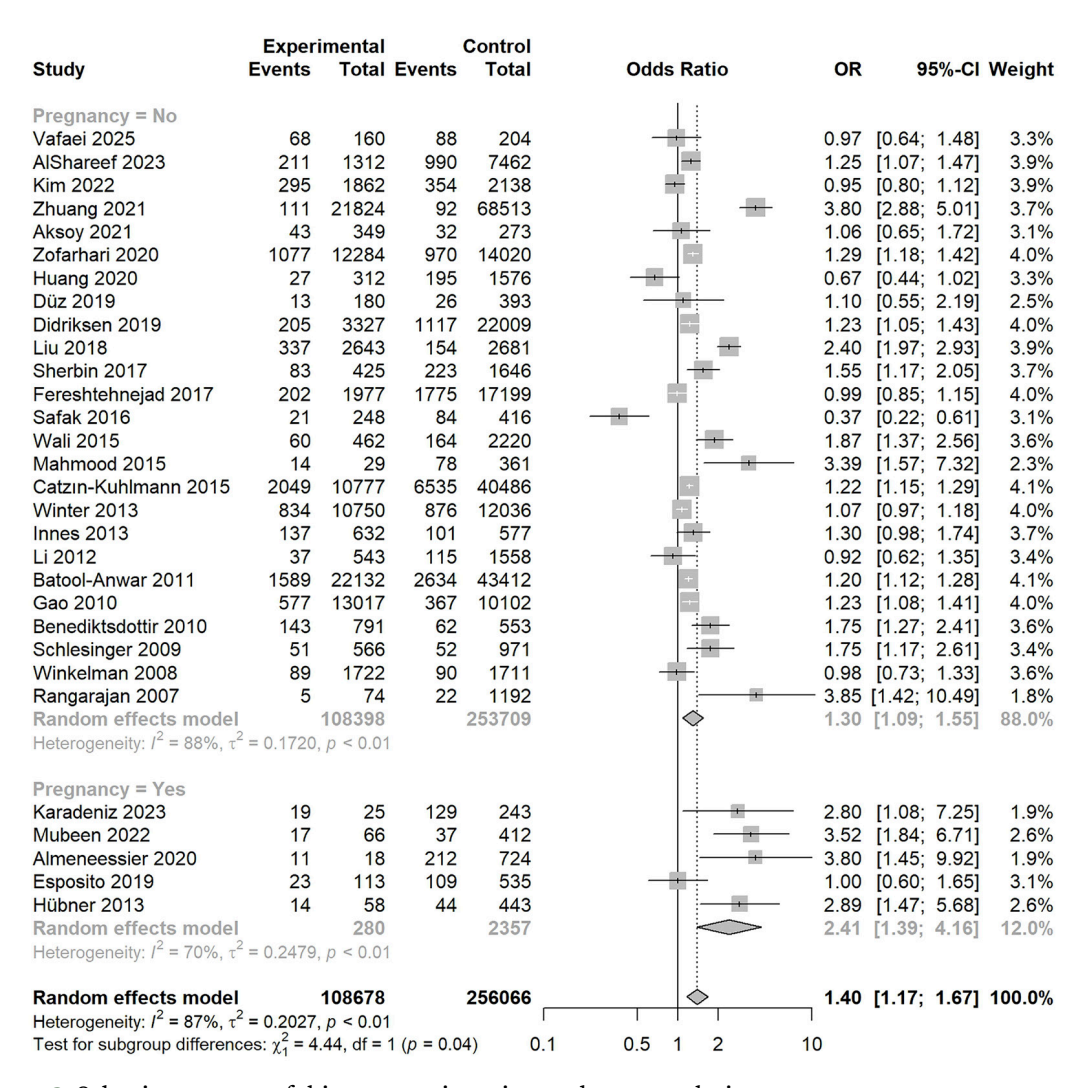
Selection process of this systematic review and meta-analysis.

We found that smoking was associated with an increased risk of RLS (OR = 1.40; 95% CI = 1.17, 1.67, *P* < 0.001; *I*^2^ = 87%) (**Figure 2**) in both the non-pregnant (OR = 1.30; 95% CI = 1.09, 1.55, *P* = 0.004; *I*^2^ = 88%) and pregnant women (OR = 2.41; 95% CI = 1.39, 4.16, *P* = 0.002; *I*^2^ = 70%), with pregnant women showing a higher risk of RLS than non-pregnant adults (*P* = 0.04) (**Figure 2**). Thirteen studies, comprising 85 933 participants (32 514 smokers and 53 419 non-smokers), have also differentiated current from former smokers [27,28,35,38,39,41–43,46–50] in the non-pregnant adults. Current smokers were associated with a significantly increased risk of RLS compared with former smokers (OR = 1.09; 95% CI = 1.02, 1.16, *P* = 0.01; *I*^2^ = 0%) (Figure S2 in the **Online Supplementary Document**).

### Sensitivity analyses, subgroup analyses and meta-regression

The results of the sensitivity analyses indicated that the smoking-RLS association remained stable when each study was omitted (Figures S3 in the **Online Supplementary Document**). However, when evaluating whether current smokers had an increased risk of RLS compared with former smokers, the significant association disappeared after removing the study by Batool-Anwar *et al.* (OR = 1.07; 95% CI = 0.99, 1.05, *P* = 0.10) (Figures S4 in the **Online Supplementary Document**) [46]. As we rated three studies as high and 27 as moderate quality, we further performed quality-based sensitivity analyses enrolling only moderate-quality studies (Figures S5 and S6 in the **Online Supplementary Document**). Although smoking was also positively associated with RLS in both the non-pregnant adults (OR = 1.36; 95% CI = 1.16, 1.60) and pregnant women (OR = 2.18; 95% CI = 1.14, 4.17), we observed no significant difference between the two groups (*P* = 0.17). Subgroup analyses suggested that multi-centre studies (*P* = 0.04) and diagnostic criteria for RLS (*P* = 0.02) could induce subgroup differences in the association between smoking and RLS (**Table 2**). When comparing risk of RLS between current and former smokers, both location (*P* = 0.58) and multi-centre studies (*P* = 0.84) did not support a subgroup difference (Table S3 in the **Online Supplementary Document**). Results of meta-regression suggested that only age (*P* = 0.02) could contribute to the heterogeneity of smoking-RLS association, and we did not observe any significant factor when comparing current with previous smokers (**Table 3**; Table S4 in the **Online Supplementary Document**).

**Table 2:** Subgroup analyses of the association between smoking and RLS

Smoking *vs.* non-smoking	Number of studies	OR (95% CI)	*I* ^2^	*P*-value
Location				0.15
*Asia*	19	1.51 (1.13, 2.02)	90.7%	
*Non-Asia*	11	1.21 (1.16, 1.28)	55.0%	
Number of centres				0.04
*Single-center*	8	1.84 (1.39, 2.44)	65.3%	
*Multi-center*	22	1.28 (1.05, 1.57)	86.7%	
Study type				0.96
*Cross-sectional*	28	1.40 (1.18, 1.67)	87.1%	
*Cohort*	2	1.36 (0.33, 5.66)	92.2%	
Diagnostic criteria of RLS				0.02
*IRLSSG 2003*	20	1.41 (1.12, 1.79)	89.7%	
*IRLSSG 2012*	6	1.78 (1.11, 2.84)	71%	
*Other questionnaires*	4	1.07 (0.95, 1.21)	42.4%	

RLS – restless leg syndrome, IRLSSG – International Restless Legs Syndrome Study Group

**Table 3:** Meta-regression of the association between smoking and RLS

Variables	Number of studies	Estimate (95% CI)	Standard error	z	*P*-value
Age	18	−0.02 (−0.04, −0.04)	0.01	−2.42	0.02
Proportion of male	22	−0.11 (−0.75, 0.53)	0.33	−0.34	0.74
BMI	11	−0.11 (−0.28, 0.07)	0.09	−1.18	0.24
Hypertension	14	−1.40 (−2.98, 0.18)	0.81	−1.74	0.08
DM	14	−2.05 (−7.38, 3.28)	2.72	−0.75	0.45
Alcohol	6	0.12 (−1.49, 1.74)	0.83	0.15	0.88
Depression	3	−6.96 (−15.00, 1.08)	4.10	−1.70	0.09
CAD	4	−6.03 (−14.50, 2.44)	4.32	−1.40	0.16
Anaemia	4	3.58 (−1.33, 8.48)	2.50	1.43	0.15

BMI – body mass index, CAD – coronary artery disease, DM – diabetes mellitus

### Asymmetry tests

Results of asymmetry tests suggested significant publication bias in the association between smoking and RLS (*P* = 0.02); however, we observed no publication bias when comparing RLS risk between current and former smokers (*P* = 0.83) (Figure S7 and S8 in the **Online Supplementary Document**).

### MR results

**Figure 3:**
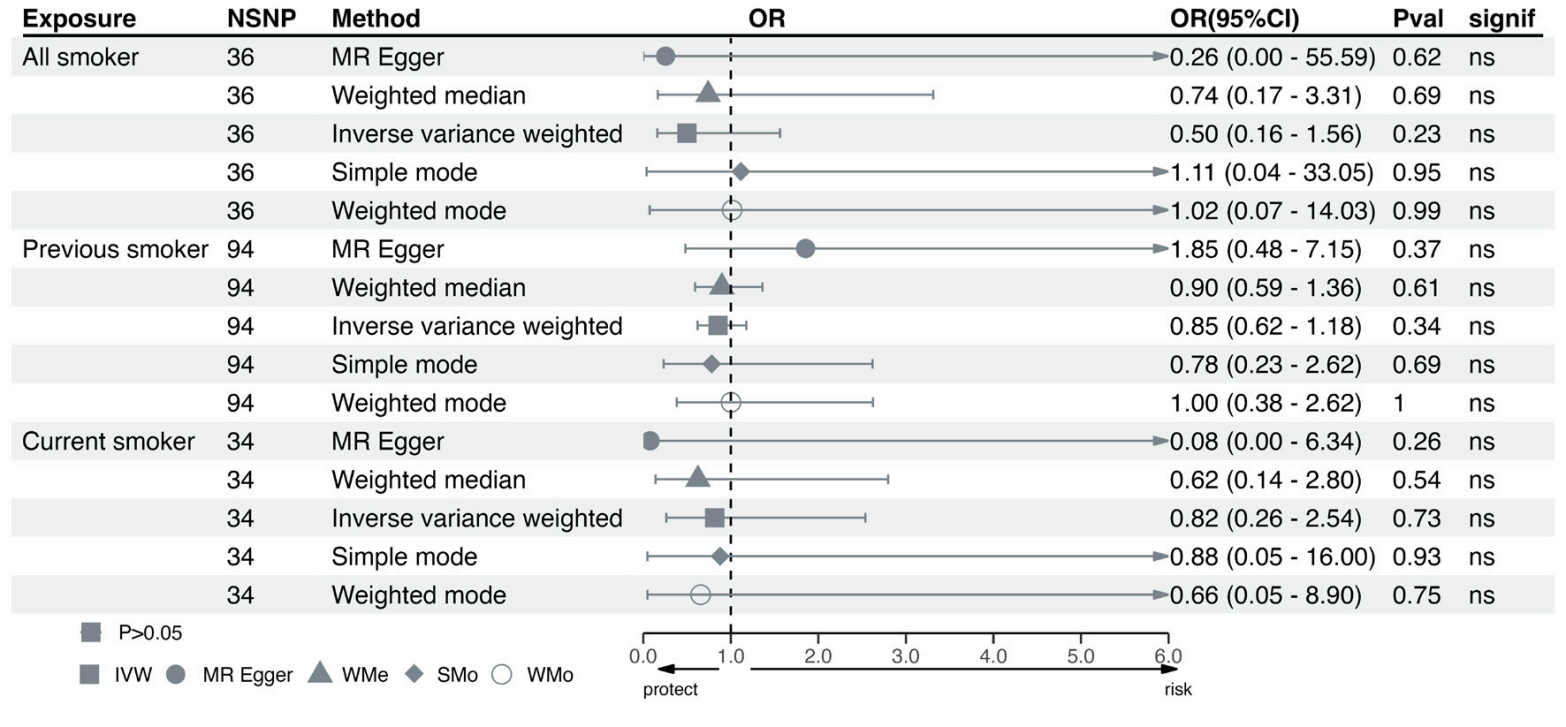
Association between smoking and RLS in the non-pregnant adults population and pregnant women.

We identified a total of 36 SNPs for ever smokers, 94 for former smokers, and 34 for current smokers (Tables S5–7 in the **Online Supplementary Document**). None of the ever smokers (OR = 0.50; 95% CI = 0.16, 1.56), former smokers (OR = 0.85; 95% CI = 0.62, 1.18, *P* = 0.34) and current smokers (OR = 0.82; 95% CI = 0.26, 2.53, *P* = 0.73) were causally linked with the risk of RLS, which was supported by all other four approaches (**Figure 3**). Results of leave-one-out analyses suggested that results remained stable after omitting each SNP (Figures S9–11 in the **Online Supplementary Document**). We detected no evidence of pleiotropy among all analyses; however, we observed significant heterogeneity in the association between previous smoking and RLS (*P* = 0.005 for MR Egger and *P* = 0.004 for IVW) (Table S8 in the **Online Supplementary Document**).

## DISCUSSION

With a total of 30 eligible studies, this systematic review and meta-analysis suggested that smoking was associated with increased risk of RLS. Pregnant women who smoked demonstrated significantly higher susceptibility to developing RLS compared with smokers in the non-pregnant adults. Moreover, compared with former smokers, we also associated current smokers with increased risk of RLS. However, further MR analyses did not support the causal association between smoking and RLS. The following factors may contribute to the discrepancy between meta-analysis and MR: we conducted the meta-analysis based on data from observational studies, which were susceptible to residual confounding, and we obtained the data source of meta-analysis and MR from different ethnic groups, and observed significant heterogeneity in both meta-analysis and MR. Further preclinical and clinical studies are still needed to investigate the biological link between smoking and RLS.

The biological mechanisms linking smoking and RLS were not fully understood. In previous studies, smoking was observed to alter the levels of iron metabolism-related proteins, thereby increasing the risk of iron dysregulation, while iron dysregulation was also observed in patients with RLS [2,52]. Moreover, smoking has been shown to promote the release of dopamine via stimulating dopaminergic neurons from both striatal and extrastriatal regions [53]. However, decreased availability of dopamine transporters was observed in both smokers and RLS patients, suggesting that smoking may lead to dopamine system dysfunction despite its promoting role [54,55]. Moreover, those with dopamine system dysfunction may continue to smoke in an attempt to stabilise or stimulate the dopamine system. A plausible hypothesis is that the dopaminergic system could serve as an important covariate in the association between smoking and RLS. Individuals with underlying instability in dopaminergic function might simultaneously exhibit a higher propensity for smoking and an increased risk of developing RLS. Additionally, smoking was associated with active inflammatory responses and elevated levels of inflammatory indicators [56,57], while elevated levels of C-reactive proteins were also observed in patients with RLS [58]. RLS could also be comorbid with multiple diseases such as depression, insomnia, or periodic limb movement disorder. These diseases could also be affected by smoking and participate in the smoking-RLS association. However, despite the shared characteristics above, few studies have investigated the mechanical link between smoking and RLS, which warrants further studies to address it.

We found that compared with the non-pregnant adults, smoking may exert a greater impact on pregnant women in developing RLS. As smoking has already been shown to become the most prevalent preventable cause of pregnancy complications, including ectopic pregnancy, preterm birth and foetal death, health education for pregnant women should place greater emphasis on the detrimental effects of smoking [59]. However, this subgroup difference disappeared after we included only moderate-quality studies. Therefore, these findings are preliminary and warrant further research. We also found that current smokers were associated with an increased risk of RLS compared to former smoking, supporting the potential value of smoking cessation in RLS prevention and treatment. A study by Mountifield first reported a 70-year-old woman with RLS who was completely relieved after smoking cessation [60]. Romigi *et al.* reported a 74-year-old woman with RLS who applied varenicline as antismoking treatment and experienced RLS amelioration afterwards [61]. However, further prospective studies or randomised controlled trials exploring the effect of smoking cessation for the prevention and treatment of RLS remain limited. Moreover, the significant effect of current smokers disappeared after we removed the study by Batoo-Anwar *et al.* from our analysis [46]. This study was conducted among middle-aged women, which could serve as a potential source of bias. Results of subgroup analyses showed that the multi-centre studies and diagnostic criteria for RLS may contribute to heterogeneity of results. Multi-centre studies were more representative than single-centre studies, and the International RLS Study Group (IRLSSG) criteria were the most authoritative diagnostic standard for RLS [62,63]. Therefore, we recommend that future multi-centre studies applying IRLSSG criteria to identify RLS investigate this topic. Additionally, age was identified as a source of heterogeneity in the meta-regression analyses. Future studies could explore the association between smoking and RLS in different age groups.

Our study had some limitations. First, we based the diagnosis of RLS and the history of smoking on subjective questionnaires, which were susceptible to potential bias. Second, due to a lack of data, it was difficult to summarise the associations between smoking and different severities of RLS, which warrant further research. We also did not explore whether smoking may affect the longitudinal progression of symptoms and mortality of RLS patients. Third, although we conducted sensitivity analyses, subgroup analyses and meta-regression, we could only partly explain the source of heterogeneity. The highly heterogeneous pooled results may affect the reliability of conclusions. Fourth, due to the heterogeneity among different data sources, the definitions of exposure and outcome may not be consistent between meta-analysis and MR, which may reduce the reliability of our results. Finally, the influence of reverse causation was hard to rule out. It is plausible that the discomfort and chronic sleep disruption caused by RLS lead individuals to initiate or increase smoking as a form of self-medication or to cope with stress and fatigue.

## CONCLUSIONS

We found that smoking was associated with increased risk of RLS in both non-pregnant adults and pregnant women, and current smokers was associated with elevated risk of RLS compared to former smokers in non-pregnant adults. However, we did observe no causal association between smoking and RLS. Future preclinical and prospective clinical studies should further explore the detailed biological link between smoking and RLS.

## Additional material


Online Supplementary Document
