# Supplementary material for: Smoking and risk of restless leg syndrome: a systematic review, meta-analysis, and Mendelian randomisation
Source: J Glob Health. 2026 Mar 20;16:04067. doi: 10.7189/jogh.16.04067 (PMC13002175; doi:10.7189/jogh.16.04067)
Supplement: Online Supplementary Document [file jogh-16-04067-s001.pdf]

Supplement to: Du D, Qin J, Tang X, Gao L, Wu Y, Chen Z, Chen F, Luo F, Shen Y. Smoking and risk of restless leg syndrome: a systematic review, meta-analysis, and Mendelian randomisation. J Glob Health. 2026;16:04067.

Supplementary materials

Figure S1 Selection process of this systematic review and meta-analysis

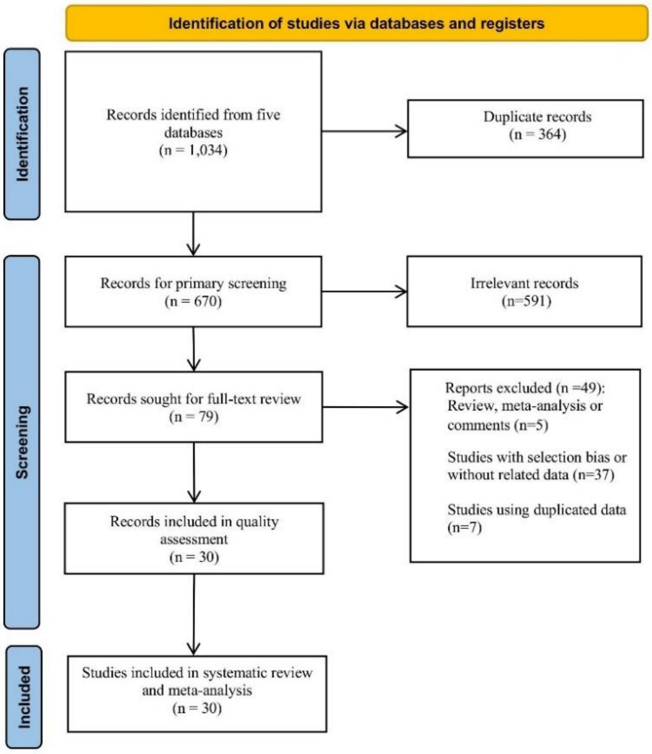

Figure S2 Current smokers were associated with increased risk of RLS compared with previous smokers

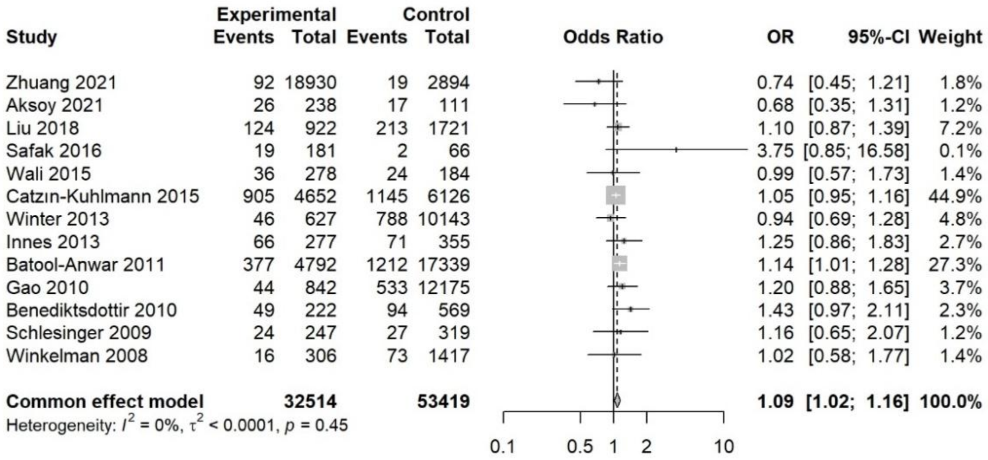

RLS: restless leg syndrome

Figure S3 Sensitivity analysis of association between smoking and RLS

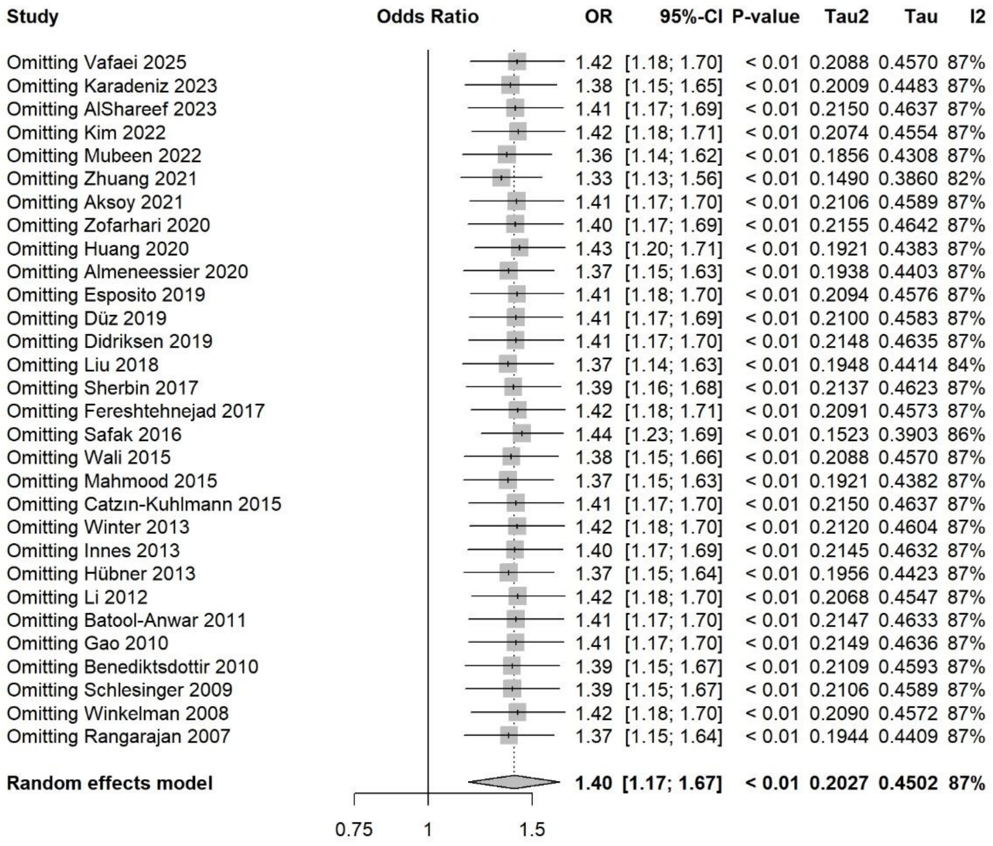

RLS: restless leg syndrome

Figure S4 Sensitivity analysis of whether current smokers increase the risk of RLS compared to previous smokers

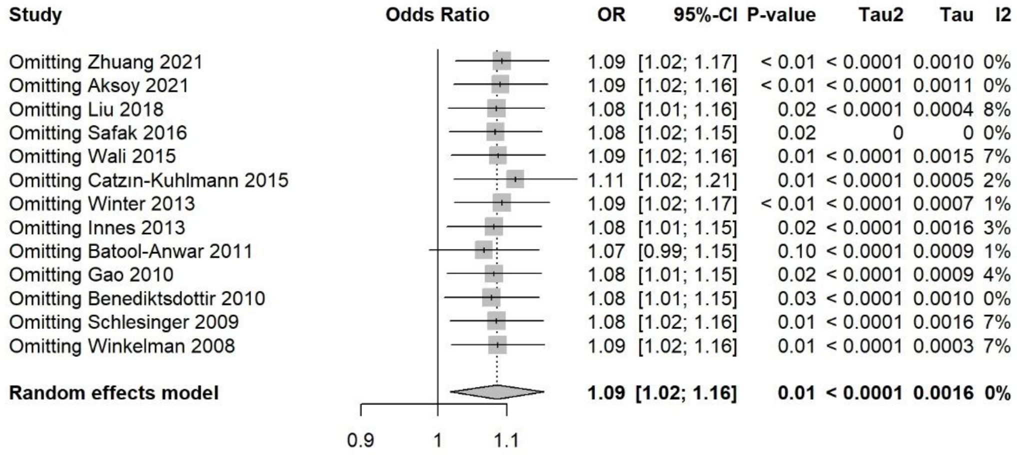

RLS: restless leg syndrome

Figure S5 Association between smoking and RLS in studies with moderate quality

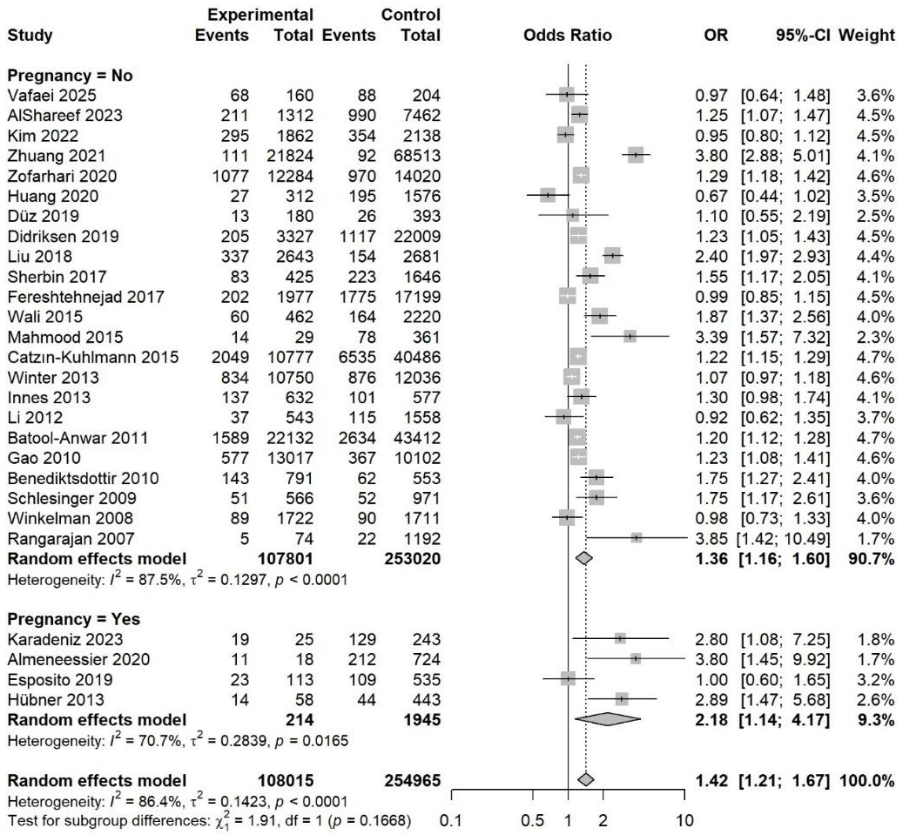

RLS: restless leg syndrome

Figure S6 Leave-one-out analysis of studies with moderate quality

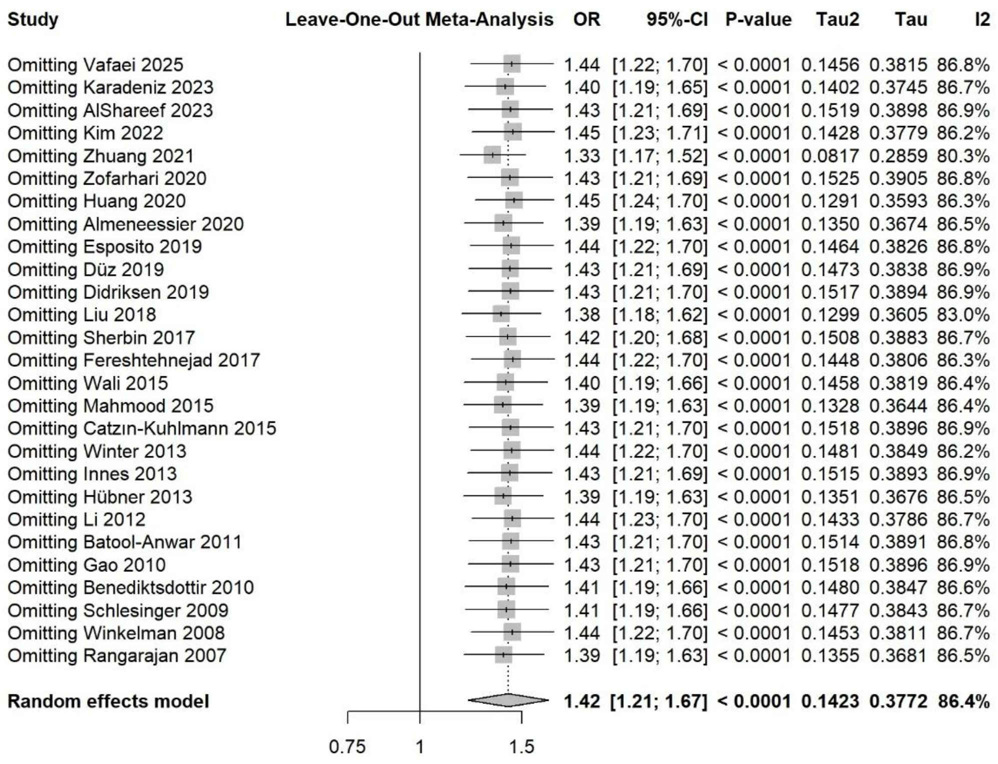

Figure S7 Publication bias of smoking-RLS association

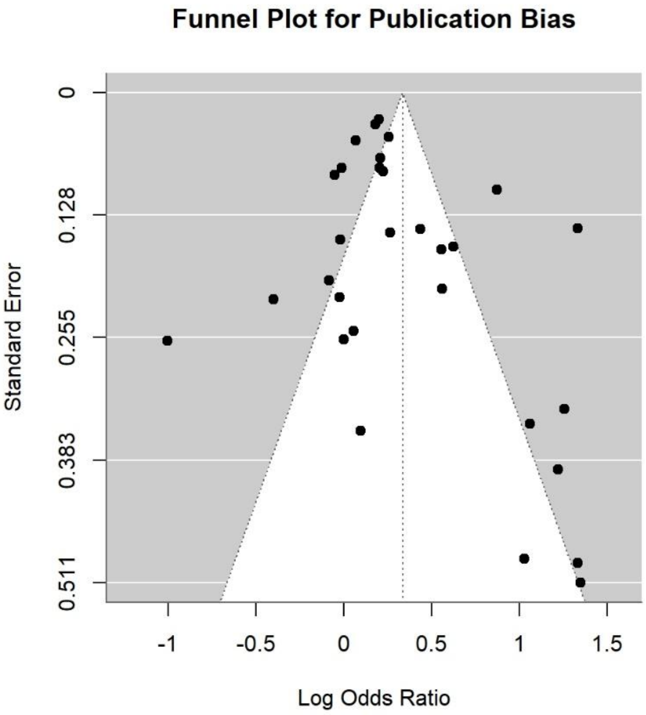

RLS: restless leg syndrome

Figure S8 Publication bias of current vs former smokers in RLS

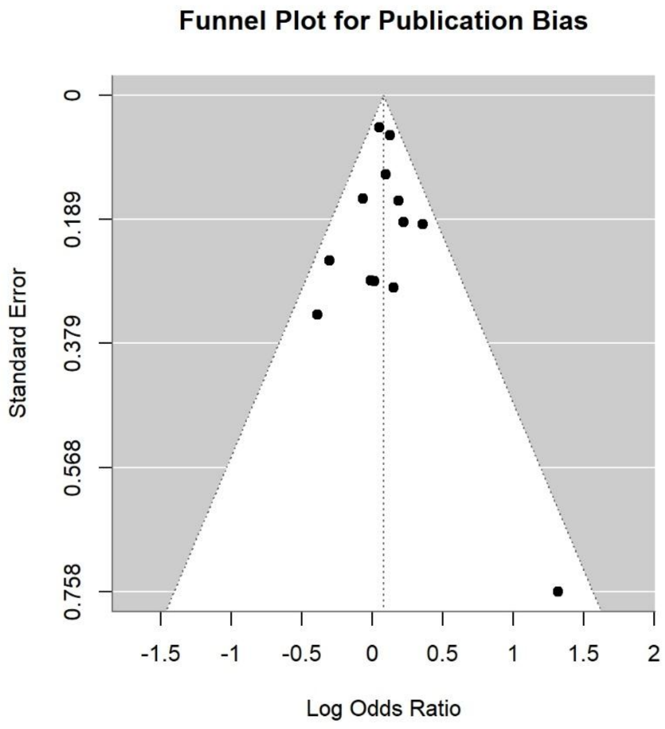

RLS: restless leg syndrome

Figure S9 Leave-one-out analysis of the causal association between ever smoking and RLS

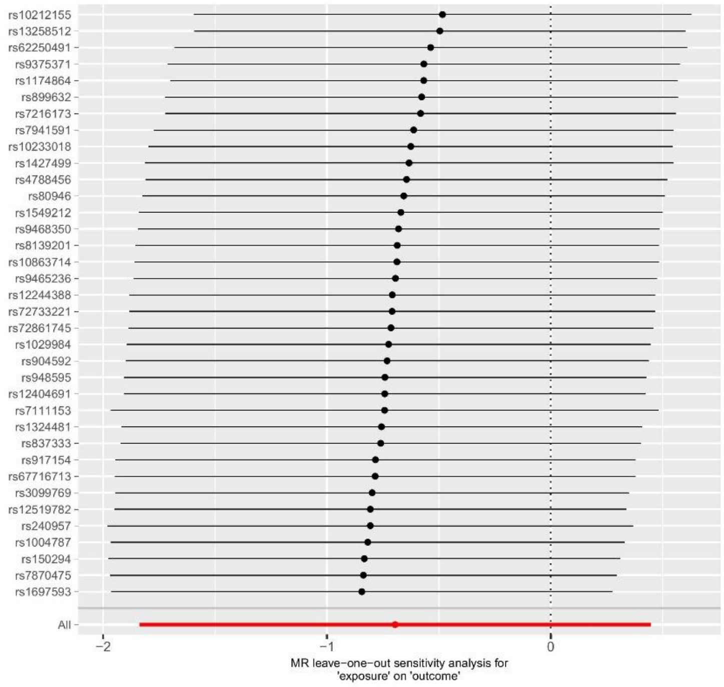

RLS: restless leg syndrome

Figure S10 Leave-one-out analysis of the causal association between previous smoking and RLS

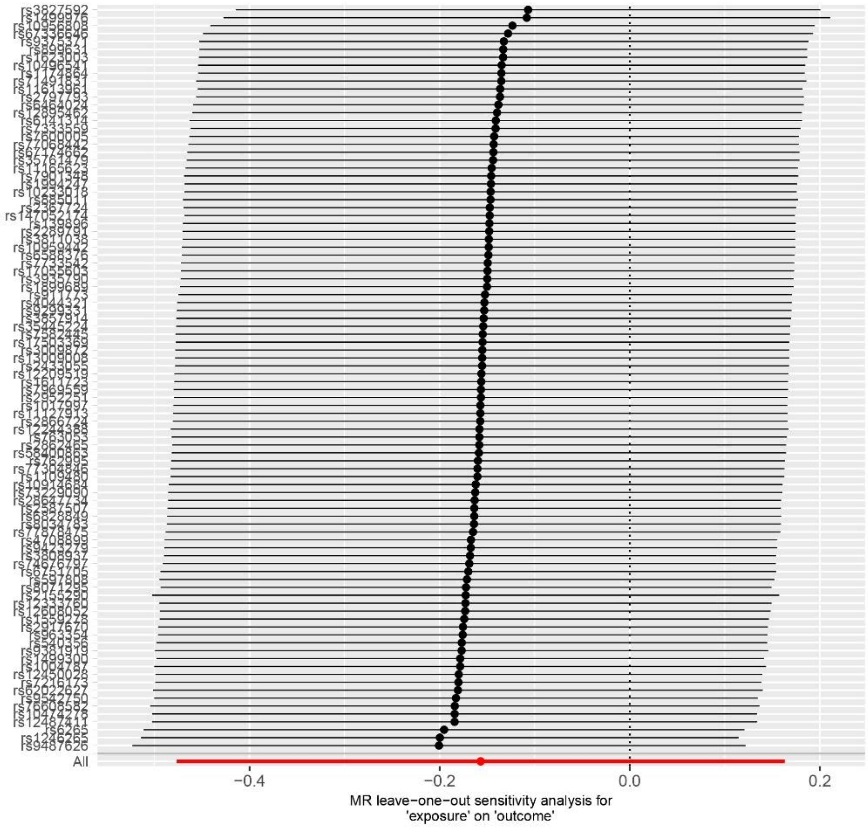

RLS: restless leg syndrome

Figure S11 Leave-one-out analysis of the causal association between previous smoking and RLS

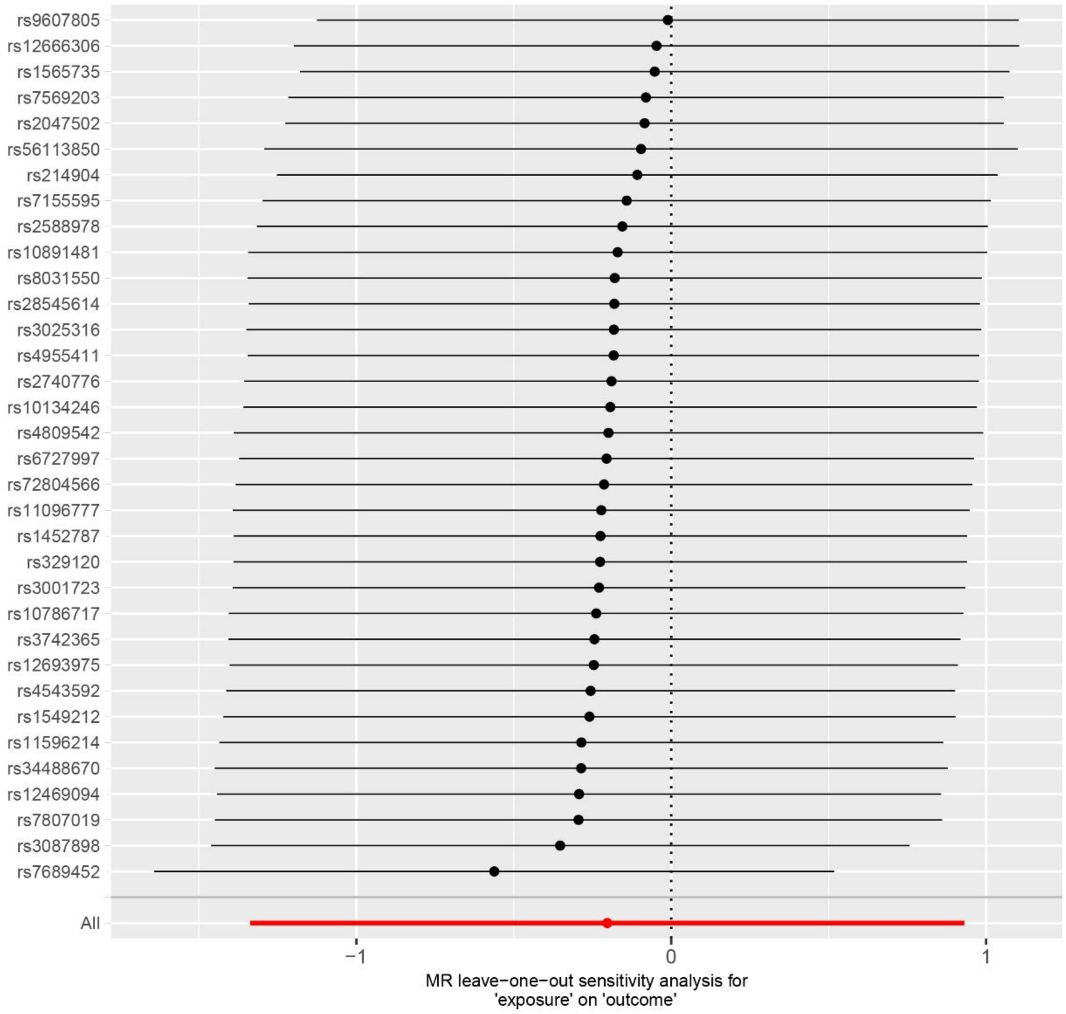

RLS: restless leg syndrome

Table S1. Outline of JoGH guideline items

JoGH guideline item	Author's Response
1. Please list all papers published by each co-author in previous 3 years that were based on secondary analysis of a big data repository	1.J Affect Disord. 2026 Jan 1;392:120151. (DD, YS and FL)<br>2. COPD. 2025 Dec;22(1):2502118. (DD, JQ, LG, YW, ZC, YS and FL)<br>3. Nat Sci Sleep. 2024 Dec 18;16:2099-2110. (DD, JQ, YS and FL)<br>4. J Psychosom Res. 2024 Dec;187:111925. (DD, YS and FL)<br>5. Int J Chron Obstruct Pulmon Dis. 2024 Jul 15;19:1649-1660. (DD, YS and FL)<br>6. Heliyon. 2024 May 18;10(10):e31524. (DD, YS and FL)<br>7. Front Public Health. 2024 Oct 2;12:1445257. (DD)<br>8. Ann Med. 2025 Dec;57(1):2522317. (JQ, YW)
2. Please explain the key elements of your study design and the use of the available datasets that make your study an original scientific contribution	The prior meta-analysis was based on observational studies and could not reach a causal conclusion. We have designed this MR study to verify whether smoking causally increased the risk of RLS and verified the findings in the meta-analysis.
3. Please list all publications that addressed similar research questions in the same dataset and indicate where you cited them in your paper	No existing publications addressed the association between smoking and RLS.
4. Please explain how you addressed multiple testing through an appropriately rigorous statistical threshold and indicate this in the methods section	We have used data of UK biobank for MR analyses. Linkage disequilibrium was evaluated with a window size of r2=0.001 and kb=10,000, and F statistics were calculated to ensure the strength of each SNPs. Only SNPs with a F>10 can be enrolled for further analysis. MR egger regression was applied to evaluate potential horizontal pleiotropy, and Cochrane's Q test was also applied for heterogeneity assessments. Leave-one-out analyses was performed to evaluate stability of results and identify outliers.
5. Please declare to what extent have AI chatbots been used in developing your paper and to which parts of the paper did they	We did not use AI.

contribute

Table S2 Full search strategies

#1 (((((((Smoking) OR (Smoking Behaviors)) OR (Behavior, Smoking)) OR (Smoking Habit)) OR (Tobacco Smoking)) OR (Cigarette Smoking)) OR (Cigar Smoking)) OR (Nicotine)
#2 (((((((Restless Legs Syndrome) OR (Restless Leg Syndrome)) OR (Syndrome, Restless Leg)) OR (Restless Legs)) OR (Willis Ekbom Disease)) OR (Willis Ekbom Syndrome)) OR (Willis-Ekbom Disease)) OR (Wittmaack Ekbom Syndrome)
#3 #1 AND #2

Table S3 Subgroup analyses of whether current smokers increased RLS risk compared to previous smokers

Subgroup	Number of studies	OR (95%CI)	I^2	p for subgroup difference
**Location**				0.58
Asia	6	1.04 (0.86, 1.24)	24.9%	
Non-Asia	7	1.09 (1.02, 1.17)	0%	
**Number of centers**				0.84
Single-center	2	1.08 (1.01, 1.16)	15.6%	
Multi-center	11	1.11 (0.89, 1.38)	0%	

RLS: restless leg syndrome

Table S4 Meta-regression of whether current smokers increased RLS risk compared to previous smokers

Variables	Number of studies (n)	Estimate	Standard error	z	p	Lower 95% CI	Upper 95% CI
Age	7	-0.004	0.01	-0.61	0.54	-0.02	0.01
Proportion of male	10	-0.07	0.10	-0.66	0.51	-0.27	0.14
BMI	6	0.16	0.59	0.28	0.78	-0.10	0.13
Hypertension	7	-0.55	0.53	-1.03	0.30	-1.59	0.50
DM	7	-4.76	2.95	-1.62	0.11	-10.54	1.02
CAD	4	-0.14	1.90	-0.07	0.94	-3.86	3.58

BMI: body mass index; DM: diabetes mellitus; CAD: coronary artery disease

Table S5 Baseline information of SNPs to explore the causal association between ever smoking and RLS.

SNP	Effect allele	Other allele	Beta exposure	Beta outcome	EAF exposure	EAF outcome	SE outcome	p outcome	SE exposure	p exposure
rs1004787	A	G	-0.00777	-0.02776	0.470083	0.625983	0.243873	0.023817	0.001197	8.54E-11
rs10212155	A	G	-0.00994	0.06812	0.148473	0.783664	0.014657	0.02791	0.001677	3.01E-09
rs10233018	G	A	-0.00845	0.022834	0.495559	0.597141	0.331687	0.023523	0.00119	1.24E-12
rs1029984	T	G	-0.00704	-0.00367	0.436638	0.597466	0.876348	0.023597	0.001215	6.78E-09
rs10863714	G	A	0.006793	-0.0072	0.423716	0.598108	0.758543	0.023436	0.001204	1.66E-08
rs1174864	A	G	-0.0067	0.0438	0.448309	0.58496	0.061187	0.023396	0.001197	2.18E-08
rs12244388	A	G	-0.00841	0.00248	0.664094	0.326961	0.919358	0.024491	0.001258	2.38E-11
rs12404691	T	A	-0.00923	-0.01241	0.838419	0.844483	0.695686	0.031727	0.001613	1.04E-08
rs12519782	G	T	0.009415	0.040924	0.856869	0.143214	0.212873	0.032852	0.001697	2.87E-08
rs1324481	G	T	-0.00722	-0.01474	0.317329	0.69537	0.555623	0.025009	0.001279	1.64E-08
rs13258512	G	A	0.00724	-0.05913	0.577462	0.492615	0.009961	0.022945	0.001205	1.90E-09
rs1427499	G	A	0.010178	-0.02103	0.288967	0.694033	0.399408	0.02496	0.00131	7.94E-15
rs150294	G	A	0.007686	0.030363	0.599394	0.430901	0.190159	0.023176	0.001217	2.72E-10
rs1549212	T	C	0.00678	-0.01304	0.37486	0.657451	0.5904	0.024229	0.001229	3.41E-08
rs1697593	T	C	-0.00654	-0.04053	0.51637	0.473738	0.078496	0.023036	0.001191	3.91E-08
rs240957	C	T	0.012242	0.019091	0.159305	0.801697	0.509154	0.028919	0.001626	5.14E-14
rs3099769	A	G	-0.0068	-0.0262	0.589237	0.407513	0.263284	0.02342	0.001216	2.22E-08
rs4788456	A	G	0.007292	-0.02066	0.679737	0.332077	0.397583	0.024423	0.001277	1.13E-08
rs62250491	T	G	0.009457	-0.04696	0.37138	0.716213	0.066176	0.025562	0.001232	1.63E-14
rs67716713	A	C	0.007732	0.017224	0.513446	0.483627	0.453568	0.022982	0.001188	7.62E-11
rs7111153	C	T	-0.01533	0.004636	0.614446	0.625556	0.847117	0.024046	0.001225	5.74E-36
rs7216173	T	A	0.008512	-0.0525	0.219258	0.174243	0.085458	0.030523	0.001456	5.08E-09
rs72733221	G	A	-0.00905	0.001997	0.836602	0.221102	0.942565	0.027715	0.001613	2.00E-08

rs72861745	T	C	0.009542	0.000246	0.7497	0.168553	0.993651	0.030918	0.001375	3.93E-12
rs7870475	C	T	-0.00701	-0.03539	0.525338	0.548466	0.125333	0.023091	0.00119	3.90E-09
rs7941591	C	T	-0.00748	0.029917	0.330229	0.670164	0.221122	0.024451	0.001264	3.35E-09
rs80946	A	G	0.007123	-0.0187	0.351309	0.733528	0.470964	0.025933	0.001253	1.29E-08
rs8139201	C	T	0.007024	-0.00827	0.669923	0.274966	0.75127	0.026096	0.001266	2.90E-08
rs837333	C	T	-0.00672	-0.01441	0.523163	0.511876	0.532156	0.023066	0.001196	1.88E-08
rs899632	C	T	0.007513	-0.03925	0.612373	0.352744	0.102863	0.024066	0.001224	8.29E-10
rs904592	T	C	-0.00676	-0.00633	0.516498	0.398523	0.788606	0.023606	0.001199	1.69E-08
rs917154	G	A	0.009065	0.021124	0.845182	0.223092	0.444004	0.027597	0.001645	3.59E-08
rs9375371	A	G	-0.00782	0.041947	0.731058	0.328852	0.086018	0.024433	0.001342	5.63E-09
rs9465236	G	A	0.006562	-0.00508	0.448446	0.657396	0.834544	0.024297	0.001197	4.15E-08
rs9468350	G	A	0.007573	-0.01477	0.752117	0.122383	0.674502	0.035161	0.001377	3.83E-08
rs948595	T	C	-0.00693	-0.00915	0.60807	0.365987	0.701126	0.023828	0.001218	1.26E-08

SNP: single nucleotide polymorphism; RLS: restless leg syndrome; EAF, effect allele frequency; CHR: chromosome; POS: position; SE: standard error.

Table S6 Baseline information of SNPs to explore the causal association between previous smoking and RLS.

SNP	Effect allele	Other allele	Beta exposure	Beta outcome	EAF exposure	EAF outcome	SE outcome	p outcome	SE exposure	p exposure
rs1004787	A	G	-0.02083	-0.02776	0.532258	0.625983	0.023817	0.243873	0.002709	1.50E-14
rs1017997	A	G	-0.01519	0.001875	0.62039	0.62527	0.023737	0.937033	0.002775	4.37E-08
rs10233018	G	A	-0.01514	0.022834	0.503043	0.597141	0.023523	0.331687	0.002696	1.93E-08
rs10474278	G	A	-0.01849	-0.05016	0.746763	0.730349	0.025999	0.053695	0.003117	2.99E-09
rs10496541	T	C	-0.01747	0.046652	0.754868	0.717402	0.025552	0.067889	0.003128	2.33E-08
rs10914684	A	G	0.016593	0.007265	0.325484	0.335799	0.024343	0.765352	0.002874	7.77E-09
rs10956808	G	T	0.018912	-0.05259	0.422089	0.496291	0.023001	0.022232	0.002737	4.89E-12

rs10959442	G	T	-0.0172	0.016562	0.46641	0.485685	0.022993	0.471347	0.002699	1.86E-10
rs1109480	A	G	0.016339	0.003869	0.389103	0.352256	0.024242	0.87319	0.002787	4.54E-09
rs11127913	C	T	0.017272	-0.00222	0.390267	0.393011	0.023547	0.924865	0.002758	3.81E-10
rs11165623	A	G	-0.01581	0.023549	0.504148	0.428696	0.023219	0.310484	0.002694	4.42E-09
rs11613961	C	T	-0.02936	0.101722	0.086782	0.054723	0.051064	0.046367	0.004797	9.33E-10
rs1174864	A	G	-0.01536	0.0438	0.54986	0.58496	0.023396	0.061187	0.002713	1.51E-08
rs12209519	G	A	-0.01589	0.004016	0.407575	0.369579	0.023865	0.866369	0.002754	7.98E-09
rs12244388	A	G	-0.02551	0.00248	0.338108	0.326961	0.024491	0.919358	0.002848	3.32E-19
rs12333760	C	T	0.021397	0.026411	0.165515	0.226892	0.02741	0.335273	0.003633	3.87E-09
rs12450028	T	C	-0.01661	-0.04094	0.345053	0.337069	0.024313	0.092213	0.002833	4.60E-09
rs1246265	C	T	-0.02092	-0.06588	0.695165	0.719024	0.02524	0.00905	0.002935	1.01E-12
rs12487411	A	G	0.016798	0.043365	0.470776	0.462802	0.023036	0.05977	0.0027	4.95E-10
rs12608052	T	C	0.016111	0.025932	0.518925	0.486428	0.023036	0.260278	0.002701	2.45E-09
rs12895462	C	T	0.020007	-0.04504	0.191374	0.176961	0.030162	0.135354	0.003444	6.30E-09
rs13009008	G	A	0.015755	-0.00558	0.672659	0.623752	0.023787	0.814582	0.002867	3.88E-08
rs139896	C	T	-0.01621	0.020284	0.64784	0.653441	0.024185	0.401626	0.00282	8.97E-09
rs147052174	T	G	-0.06039	0.134917	0.018556	0.008772	0.12199	0.268743	0.009974	1.40E-09
rs1499300	C	A	0.021794	0.051863	0.157852	0.14655	0.032447	0.10995	0.003694	3.64E-09
rs1499976	C	T	-0.03143	0.067931	0.852678	0.783691	0.027911	0.014938	0.003819	1.90E-16
rs1559278	C	T	0.015486	0.031409	0.361217	0.36441	0.023899	0.188776	0.002805	3.38E-08
rs1611723	G	A	0.015841	-0.00389	0.405117	0.293246	0.025362	0.878192	0.002739	7.32E-09
rs1623003	T	C	-0.01847	0.042831	0.664037	0.656266	0.024237	0.077195	0.002866	1.15E-10
rs17055603	A	G	0.016507	-0.01653	0.313692	0.327462	0.024485	0.499657	0.002912	1.43E-08
rs17503369	C	T	0.021636	-0.01086	0.182208	0.099453	0.038913	0.780204	0.003503	6.58E-10
rs1899689	T	C	-0.01542	0.015043	0.389116	0.401115	0.023457	0.521333	0.002762	2.35E-08
rs1994247	T	G	-0.01577	0.021888	0.525497	0.526954	0.023005	0.341378	0.002697	5.01E-09

rs2155290	G	C	-0.03946	-0.00544	0.38361	0.375126	0.023955	0.820331	0.00277	4.57E-46
rs2289791	T	G	0.019085	-0.02312	0.247319	0.210851	0.028342	0.414706	0.003146	1.30E-09
rs2367724	T	C	0.016007	-0.02164	0.673602	0.673605	0.024458	0.376352	0.00287	2.44E-08
rs2433055	G	T	0.014837	-0.00538	0.447651	0.429276	0.023262	0.817111	0.002715	4.62E-08
rs2587507	C	T	0.015586	0.009714	0.505702	0.512363	0.023036	0.673239	0.002678	5.89E-09
rs2797793	C	T	0.015271	-0.03989	0.603851	0.50999	0.023004	0.082909	0.002757	3.04E-08
rs2862465	A	G	0.015842	0.000296	0.409159	0.377849	0.023712	0.990048	0.002739	7.28E-09
rs28647734	A	G	-0.01917	-0.01343	0.210012	0.171077	0.030574	0.660446	0.003327	8.26E-09
rs2866724	G	A	-0.0188	0.002179	0.266293	0.318741	0.024709	0.929722	0.003053	7.28E-10
rs2917670	C	T	0.016755	0.030061	0.610217	0.611309	0.023548	0.201748	0.002762	1.32E-09
rs2952251	G	A	-0.01753	0.003354	0.739866	0.77335	0.027591	0.903248	0.003079	1.24E-08
rs3009872	C	T	0.015511	-0.00579	0.435244	0.384366	0.023667	0.806672	0.002725	1.25E-08
rs35445224	C	T	-0.02062	0.009555	0.181004	0.181717	0.029932	0.749566	0.003576	8.06E-09
rs35761479	A	G	0.022965	-0.03149	0.120751	0.173394	0.030392	0.30008	0.004136	2.82E-08
rs3808937	T	C	-0.02055	-0.02455	0.207824	0.165583	0.031097	0.429881	0.003323	6.23E-10
rs3811038	C	T	-0.01705	0.022991	0.275964	0.23019	0.027436	0.402032	0.003029	1.80E-08
rs3827592	A	G	0.017722	-0.0866	0.350953	0.387123	0.023676	0.000255	0.00283	3.78E-10
rs3857914	C	T	-0.02015	0.00878	0.302135	0.271965	0.025902	0.734636	0.002959	9.63E-12
rs3935790	A	G	-0.01547	0.015503	0.416104	0.407609	0.023421	0.50803	0.002738	1.60E-08
rs4044321	G	A	0.01837	-0.00963	0.641902	0.661155	0.024304	0.692017	0.002816	6.87E-11
rs4708899	G	A	0.015898	0.016063	0.575572	0.563189	0.023365	0.491776	0.002735	6.17E-09
rs540356	A	C	-0.01845	-0.02839	0.413629	0.411389	0.023397	0.224896	0.00276	2.36E-11
rs58400863	A	G	0.017627	0.000338	0.341566	0.354077	0.024028	0.988777	0.002853	6.46E-10
rs597808	G	A	0.022118	0.015316	0.515766	0.586614	0.023381	0.512439	0.002701	2.63E-16
rs6141314	A	G	-0.01916	0.028872	0.241567	0.345885	0.024209	0.233022	0.003168	1.47E-09
rs62022627	G	A	0.018963	0.033305	0.400649	0.413252	0.023317	0.153189	0.002755	5.91E-12

rs6265	T	C	0.026146	0.074839	0.189039	0.153986	0.031832	0.018722	0.00344	2.96E-14
rs6464024	T	C	0.017555	-0.03306	0.427444	0.444782	0.023154	0.153327	0.002723	1.14E-10
rs6588376	A	G	-0.01883	0.022272	0.208312	0.196858	0.028891	0.440765	0.003318	1.38E-08
rs67174662	G	A	0.016656	-0.02584	0.375322	0.413502	0.02331	0.267614	0.002786	2.26E-09
rs67336646	A	T	0.023643	-0.04562	0.626947	0.716292	0.025512	0.073746	0.002781	1.85E-17
rs6751705	G	T	-0.02247	-0.01262	0.512137	0.49534	0.02305	0.58408	0.002693	7.11E-17
rs6828849	T	A	0.017917	0.00894	0.418816	0.341933	0.024205	0.711885	0.002734	5.65E-11
rs71491831	A	G	0.030574	-0.06013	0.075719	0.099693	0.038348	0.116856	0.0051	2.04E-09
rs7216173	T	A	0.020776	0.052495	0.782099	0.825757	0.030523	0.085458	0.003309	3.43E-10
rs73229090	A	C	0.028878	0.011585	0.117859	0.094557	0.039265	0.767957	0.004228	8.43E-12
rs7333559	A	G	0.019966	-0.04379	0.788658	0.836405	0.031109	0.159257	0.00332	1.82E-09
rs74676797	A	G	-0.02335	-0.02284	0.80811	0.837381	0.031287	0.465446	0.003527	3.60E-11
rs7582445	C	A	-0.01722	0.006459	0.587784	0.62267	0.023825	0.786316	0.002738	3.16E-10
rs7600005	A	G	0.015608	-0.03676	0.373357	0.250858	0.026643	0.167696	0.002803	2.56E-08
rs762995	G	A	0.014873	0.002982	0.535061	0.473617	0.022996	0.896827	0.002702	3.71E-08
rs763053	C	T	0.019489	-0.00082	0.226141	0.349058	0.0241	0.972746	0.003236	1.72E-09
rs76608582	A	C	0.042972	0.075099	0.047264	0.057101	0.049436	0.128732	0.006667	1.15E-10
rs77068442	G	A	-0.02391	0.048589	0.107228	0.10359	0.038372	0.205414	0.004357	4.06E-08
rs77304846	C	T	0.020858	0.004293	0.183007	0.182065	0.029872	0.885713	0.003482	2.08E-09
rs7733542	G	A	-0.01566	0.016977	0.629381	0.64544	0.024022	0.479721	0.002811	2.53E-08
rs77878475	A	T	0.033082	0.014793	0.084274	0.097174	0.039378	0.707164	0.00503	4.82E-11
rs7901348	G	T	0.019295	-0.01921	0.552343	0.527669	0.023048	0.404477	0.002729	1.55E-12
rs7969559	G	A	0.017342	-0.00319	0.720515	0.64432	0.024027	0.8944	0.003001	7.49E-09
rs8034783	T	C	-0.02825	-0.01431	0.101047	0.104748	0.037581	0.703312	0.004494	3.28E-10
rs8071295	A	C	0.022507	0.034774	0.157348	0.148939	0.032463	0.284085	0.003716	1.39E-09
rs885011	C	T	0.020272	-0.01763	0.501337	0.541944	0.023047	0.444415	0.002695	5.43E-14

rs899631	T	G	0.017651	-0.04383	0.391204	0.359486	0.023949	0.067206	0.002768	1.81E-10
rs911773	C	A	0.015256	-0.01101	0.489521	0.437358	0.023215	0.63536	0.002694	1.49E-08
rs9299331	C	T	-0.01749	0.008733	0.526134	0.489065	0.022952	0.703574	0.002699	9.09E-11
rs9375371	A	G	-0.0199	0.041947	0.269381	0.328852	0.024433	0.086018	0.003044	6.27E-11
rs9381919	T	G	0.027063	0.029918	0.103581	0.185759	0.029591	0.311994	0.004425	9.57E-10
rs9423279	G	C	0.016448	0.016013	0.657058	0.598522	0.023552	0.49656	0.002892	1.29E-08
rs9487626	T	C	0.032465	0.040252	0.817372	0.713434	0.025413	0.113214	0.003483	1.16E-20
rs9542750	C	T	0.015155	0.046468	0.585734	0.518245	0.023145	0.044669	0.002751	3.61E-08
rs963354	A	C	-0.01764	-0.03266	0.67342	0.691306	0.024968	0.19088	0.002877	8.65E-10

SNP: single nucleotide polymorphism; RLS: restless leg syndrome; EAF, effect allele frequency; CHR: chromosome; POS: position; SE: standard error.

Table S7 Baseline information of SNPs to explore the causal association between current smoking and RLS.

SNP	Effect allele	Other allele	Beta exposure	Beta outcome	EAF exposure	EAF outcome	SE outcome	p outcome	SE exposure	p exposure
rs10134246	A	G	0.0115497	-0.00711036	0.909375	0.918382	0.0420552	0.86574	0.00199674	7.28E-09
rs10786717	G	A	0.00770944	0.00751486	0.329048	0.324689	0.0245468	0.759495	0.00121047	1.90E-10
rs10891481	G	A	0.00845303	-0.00846161	0.383657	0.483105	0.0230955	0.714085	0.00117365	5.92E-13
rs11096777	C	T	0.00945455	0.00359297	0.18038	0.198833	0.0287753	0.900632	0.00148135	1.74E-10
rs11596214	A	G	-0.00641136	-0.021363	0.406984	0.474883	0.0230756	0.35456	0.00116851	4.09E-08

rs12469094	C	A	-0.00685575	-0.0224421	0.68186	0.602109	0.0234889	0.339357	0.00122457	2.16E-08
rs12666306	G	A	-0.00920045	0.031733	0.498932	0.426895	0.0233164	0.173523	0.00114399	8.81E-16
rs12693975	A	G	0.0079645	0.0171041	0.813989	0.851403	0.0323092	0.596535	0.00146069	4.96E-08
rs1452787	G	A	0.00705403	0.00492867	0.276265	0.305153	0.0249844	0.843616	0.00127089	2.85E-08
rs1549212	T	C	-0.00751493	-0.0130416	0.626264	0.657451	0.0242292	0.5904	0.00117745	1.74E-10
rs1565735	A	T	-0.0101287	0.0543111	0.203149	0.146527	0.0326749	0.0964806	0.00142501	1.18E-12
rs2047502	A	C	0.00713317	-0.0316878	0.411335	0.401838	0.0235102	0.177714	0.00116004	7.79E-10
rs214904	T	C	-0.00657698	0.0282314	0.398277	0.382837	0.0236832	0.233244	0.0011636	1.58E-08
rs2588978	C	T	-0.00651265	0.0142794	0.519271	0.469746	0.0231156	0.536749	0.00113776	1.04E-08
rs2740776	T	C	-0.00720834	0.00513378	0.727232	0.67707	0.0245086	0.834082	0.0012828	1.92E-08
rs28545614	T	C	0.00893084	-0.0125342	0.156729	0.1186	0.0360067	0.72776	0.00157156	1.33E-08
rs3001723	A	G	0.00721497	0.00679886	0.301175	0.260585	0.0261659	0.79499	0.00123869	5.72E-09
rs3025316	C	T	0.0196756	-0.0186461	0.11429	0.0329856	0.0648222	0.773615	0.00179265	5.00E-28
rs3087898	A	G	-0.00641507	-0.0422794	0.424573	0.38067	0.0237219	0.0747016	0.00115214	2.58E-08
rs329120	T	C	-0.00666687	-0.00479797	0.419332	0.424568	0.0232869	0.836762	0.00115429	7.66E-09
rs34488670	C	T	0.00979043	0.019088	0.21112	0.222815	0.0276116	0.489375	0.00139999	2.69E-12
rs3742365	C	T	0.00667236	0.00954904	0.404833	0.437103	0.0231767	0.680332	0.0011627	9.54E-09
rs4543592	C	T	0.00629793	0.0142023	0.480069	0.39319	0.0235535	0.546524	0.00114096	3.39E-08
rs4809542	G	C	0.0167647	-0.00429248	0.069874	0.113737	0.0360665	0.905263	0.00223905	7.02E-14
rs4955411	G	A	-0.00751747	0.00923411	0.778828	0.809439	0.0294284	0.753687	0.00137006	4.09E-08
rs56113850	C	T	-0.0126594	0.0172479	0.577585	0.564105	0.0236479	0.46578	0.0011517	4.18E-28
rs6727997	G	A	0.00737776	-0.000835084	0.654335	0.699598	0.0251523	0.973514	0.00120444	9.04E-10
rs7155595	C	A	0.00695048	-0.0198582	0.325737	0.302561	0.0250785	0.428453	0.00121953	1.20E-08
rs72804566	T	A	0.0172183	0.0021369	0.03574	0.0493806	0.0532834	0.96801	0.00307032	2.05E-08
rs7569203	C	A	0.00691663	-0.0337692	0.311269	0.400549	0.0235088	0.150874	0.00123291	2.02E-08
rs7689452	G	A	-0.00742195	-0.0907263	0.31352	0.36216	0.0240587	0.000162566	0.00122923	1.56E-09

rs7807019	G	A	0.00745097	0.0205525	0.459691	0.581098	0.0233821	0.379409	0.00114226	6.89E-11
rs8031550	A	G	-0.00797846	0.008485	0.240382	0.247973	0.0265646	0.749414	0.00133233	2.12E-09
rs9607805	T	C	0.00825078	-0.0470698	0.726456	0.667958	0.0243919	0.0536402	0.00128174	1.22E-10

SNP: single nucleotide polymorphism; RLS: restless leg syndrome; EAF, effect allele frequency; CHR: chromosome; POS: position; SE: standard error.

Table S8 Results of pleiotropy and heterogeneity tests

Exposure	Outcome	Pleiotropy test			Heterogeneity test					
		MR-Egger			MR-Egger			Inverse variance weighted		
		Intercept	SE	p	Q	Q_df	p	Q	Q_df	p
Ever smoking	RLS	0.006	0.023	0.804	45.29	34	0.093	45.378	35	0.112
Previous smoking	RLS	-0.015	0.013	0.250	131.015	92	0.005	132.924	93	0.004
Current smoking	RLS	0.020	0.018	0.287	36.125	32	0.282	37.448	33	0.272

RLS: restless leg syndrome

The PRISMA checklist

Section and Topic	Item #	Checklist item	Location where item is reported
**TITLE**			
Title	1	Identify the report as a systematic review.	Page 1
**ABSTRACT**			
Abstract	2	See the PRISMA 2020 for Abstracts checklist.	Page 2-3
**INTRODUCTION**			
Rationale	3	Describe the rationale for the review in the context of existing knowledge.	Page 4-5
Objectives	4	Provide an explicit statement of the objective(s) or question(s) the review addresses.	Page 4-5
**METHODS**			
Eligibility criteria	5	Specify the inclusion and exclusion criteria for the review and how studies were grouped for the syntheses.	Page 5
Information sources	6	Specify all databases, registers, websites, organisations, reference lists and other sources searched or consulted to identify studies. Specify the date when each source was last searched or consulted.	Page 5
Search strategy	7	Present the full search strategies for all databases, registers and websites, including any filters and limits used.	Supplementary materials
Selection process	8	Specify the methods used to decide whether a study met the inclusion criteria of the review, including how many reviewers screened each record and each report retrieved, whether they worked independently, and if applicable, details of automation tools used in the process.	Page 5-6
Data collection process	9	Specify the methods used to collect data from reports, including how many reviewers collected data from each report, whether they worked independently, any processes for obtaining or confirming data from study investigators, and if applicable, details of automation tools used in the process.	Page 6
Data items	10a	List and define all outcomes for which data were sought. Specify whether all results that were compatible with each outcome domain in each	Page 6-7

Section and Topic	Item #	Checklist item	Location where item is reported
		study were sought (e.g. for all measures, time points, analyses), and if not, the methods used to decide which results to collect.	
	10b	List and define all other variables for which data were sought (e.g. participant and intervention characteristics, funding sources). Describe any assumptions made about any missing or unclear information.	Page 6-7
Study risk of bias assessment	11	Specify the methods used to assess risk of bias in the included studies, including details of the tool(s) used, how many reviewers assessed each study and whether they worked independently, and if applicable, details of automation tools used in the process.	Page 6
Effect measures	12	Specify for each outcome the effect measure(s) (e.g. risk ratio, mean difference) used in the synthesis or presentation of results.	Page 6-7
Synthesis methods	13a	Describe the processes used to decide which studies were eligible for each synthesis (e.g. tabulating the study intervention characteristics and comparing against the planned groups for each synthesis (item #5)).	Page 6-7
	13b	Describe any methods required to prepare the data for presentation or synthesis, such as handling of missing summary statistics, or data conversions.	Page 6-7
	13c	Describe any methods used to tabulate or visually display results of individual studies and syntheses.	Page 6-7
	13d	Describe any methods used to synthesize results and provide a rationale for the choice(s). If meta-analysis was performed, describe the model(s), method(s) to identify the presence and extent of statistical heterogeneity, and software package(s) used.	Page 6-7
	13e	Describe any methods used to explore possible causes of heterogeneity among study results (e.g. subgroup analysis, meta-regression).	Page 6-7
	13f	Describe any sensitivity analyses conducted to assess robustness of the synthesized results.	Page 6-7
Reporting bias assessment	14	Describe any methods used to assess risk of bias due to missing results in a synthesis (arising from reporting biases).	N
Certainty assessment	15	Describe any methods used to assess certainty (or confidence) in the body of evidence for an outcome.	Page 6-7

Section and Topic	Item #	Checklist item	Location where item is reported
**RESULTS**			
Study selection	16a	Describe the results of the search and selection process, from the number of records identified in the search to the number of studies included in the review, ideally using a flow diagram.	Page 8-9
	16b	Cite studies that might appear to meet the inclusion criteria, but which were excluded, and explain why they were excluded.	N
Study characteristics	17	Cite each included study and present its characteristics.	Table 1
Risk of bias in studies	18	Present assessments of risk of bias for each included study.	Table 1
Results of individual studies	19	For all outcomes, present, for each study: (a) summary statistics for each group (where appropriate) and (b) an effect estimate and its precision (e.g. confidence/credible interval), ideally using structured tables or plots.	Page 9-10
Results of syntheses	20a	For each synthesis, briefly summarise the characteristics and risk of bias among contributing studies.	Page 9-10
	20b	Present results of all statistical syntheses conducted. If meta-analysis was done, present for each the summary estimate and its precision (e.g. confidence/credible interval) and measures of statistical heterogeneity. If comparing groups, describe the direction of the effect.	Page 9-10, Figure 3-4
	20c	Present results of all investigations of possible causes of heterogeneity among study results.	Page 9-10
	20d	Present results of all sensitivity analyses conducted to assess the robustness of the synthesized results.	Page 9-10
Reporting biases	21	Present assessments of risk of bias due to missing results (arising from reporting biases) for each synthesis assessed.	N
Certainty of evidence	22	Present assessments of certainty (or confidence) in the body of evidence for each outcome assessed.	N
**DISCUSSION**			

Section and Topic	Item #	Checklist item	Location where item is reported
Discussion	23a	Provide a general interpretation of the results in the context of other evidence.	Page 11
	23b	Discuss any limitations of the evidence included in the review.	Page 13
	23c	Discuss any limitations of the review processes used.	Page 13
	23d	Discuss implications of the results for practice, policy, and future research.	Page 11-13
**OTHER INFORMATION**			
Registration and protocol	24a	Provide registration information for the review, including register name and registration number, or state that the review was not registered.	Page 5
	24b	Indicate where the review protocol can be accessed, or state that a protocol was not prepared.	N
	24c	Describe and explain any amendments to information provided at registration or in the protocol.	N
Support	25	Describe sources of financial or non-financial support for the review, and the role of the funders or sponsors in the review.	Page 14
Competing interests	26	Declare any competing interests of review authors.	Page 14
Availability of data, code and other materials	27	Report which of the following are publicly available and where they can be found: template data collection forms; data extracted from included studies; data used for all analyses; analytic code; any other materials used in the review.	Page 14-15

From: Page MJ, McKenzie JE, Bossuyt PM, Boutron I, Hoffmann TC, Mulrow CD, et al. The PRISMA 2020 statement: an updated guideline for reporting systematic reviews. BMJ 2021;372:n71. doi: 10.1136/bmj.n71. This work is licensed under CC BY 4.0. To view a copy of this license, visit https://creativecommons.org/licenses/by/4.0/
